# Scraps

**Published:** 1894-12

**Authors:** 


### SCRAPS

PICKED UP BY THE ASSISTANT-EDITOR.

**Clinical Records** (9).—Doctor: "You must avoid all excitement, give up all stimulants, and drink only water." Patient: "But, doctor, the idea of drinking water excites me more than anything else!"

**As Others See Us.**—"The other day I heard an apophthegm," writes Mr. James Payn, "which left me with a very exalted idea of the gentleman who uttered it. We were talking of the medical profession, and he observed of it: 'There is a great difference between a good doctor and a bad one, but very little between a good doctor and none at all.' The cynicism of the remark was deplorable, but its intelligence was indisputable."

**Coffee.**—En 1711, le *Mercure galant* publiait, sur l'air du *Noël des Bourgeois de Chastres*, une *Chanson sur le café.* Quelques jours après, on la chantait, modifiée et considérablement augmentée. On y lisait, entre autres vers mirlitonesques, les suivants, qui ont au moins le mérite de résumer les indications thérapeutiques de la boisson qui faisait alors fureur:

Si vous voulez sans peine
Vivre en bonne santé,
Sept jours de la semaine,
Prenez de bon café.
Il vous préservera de toute maladie,
Sa vertu chassera, la, la
Migraine et fluxion. don, don,
Rhume et mélancolie.

Sa force est sans égale
Contre les maux de cœur,
La glande pinéale
Y trouve sa vigueur.
Quand on y met du lait, il guérit la poitrine,
Au sang il donnera, la, la
La circulation, don, don,
Dans toute la machine.

Et cela continuait sur ce ton fluidifiant, et la chanson comptait au moins deux bonnes douzaines de couplets! . . .—*Gazette de Gynécologie.*

**A Therapeutic Retroversion.**—Much fun has been made of John of Gaddesden—one of the authorities of Chaucer's "Doctour of Physik" (see *Journal*, p. 160)—for saying that the best treatment of small-pox was to wrap the patient in red cloth, and to have everything red about the bed.

*The American Practitioner and News*, after quoting Gaddesden's directions from his *Rosa Medicinæ* and also Sir Thomas Watson's comments thereon, goes on to say: "In the light of these historical reminiscences the following, which we clip from the New York *Medical Record* is most interesting:

'RED LIGHT FOR SMALLPOX.—Finsen (*Hosp. Tid.*, No. 27, 1893) has made some observations on the effects of light on the skin. He referred to the good results obtained by Black and others by the exclusion of daylight in the treatment of smallpox, but argued that, as Widmark has shown that it is the ultra-violet rays which have the strong chemical action, it is not necessary to exclude the daylight, but by using red curtains tightly drawn, or red window panes, the injurious effects of the light can be prevented. The correctness of this hypothesis was proven by Svendsen, of Bergen, who last summer treated four cases of smallpox in unvaccinated patients by covering the windows with thick red woollen curtains. The patients escaped the suppurative stage; there was no rise of temperature, no œdema. The patients passed from the vesicular stage, which was slightly prolonged, into convalescence, and escaped scarring.'

"In this connection the verse of old John of Gaddesden's contemporary, Chaucer, is apt indeed:

'For out of the old fieldes, as men saithe,
Cometh all this new corn fro yere to yere;
And out of old bookes, in good faithe,
Cometh all this new science that men lere.'

"The moral of this is, that inasmuch as things are found to be so by experience long before science comes and tells us why they are so, much that seems silly in the practice of olden times may be found by science to have good foundation in fact.

"The old masters in music made all their wonderful blendings of melody, harmony, and orchestration long before the physicists knew why they wrote as they did, and, while the analogy is incomplete, we may expect medical science to justify much of the practice of the fathers in medicine.

"The solar spectrum tells us why John of Gaddesden made things red around the smallpox patient; bacteriology (asepsis) tells why the traditional midwife burns the hole in the compress that goes over the cord of the new-born infant; the marvellous effects of thyroid gland injected or ingested in myxœdema tell us how results may have been obtained through the administration of the viscera or glands of various animals in some affections; while hypnotism, to say nothing of other phases of psychic science, may yet redeem from scorn the superstitious belief of the ancients in stella strokes, demoniacal possession, doubles, ghosts, and witchcraft, and their faith in the therapeutic efficacy of charms, characts, amulets, fasts, sacred verses, and prayers."

**Medical Philology** (XII).—The term "Brawn" is now rarely used except in its restricted application to a dish usually prepared from pig's-head, but "brawny" in the sense of "muscular" is not uncommon. One of the most familiar instances in literature is in Longfellow's *Village Blacksmith*. Although in Middle English "brawn" was often used specifically to denote boar's flesh, it also signified any muscular structure. In an English vocabulary (of the 15th century) Brawne is given as the English equivalent of *musculus*, *sura*, and *pulpa*. (Wright's *Vocab.*, 635, 24, 25, 26.) The *Promptorium* gives "**Brawne** of a bore. *Aprina*." Derived from the *Campus Florum*, it added: "**Brawne** of a checun or cheken. *Pulpa*;" and "**Brawne** of mannys leggys or armys. *Musculus*, *lacertus*, *pulpa*." Mr. Way's note is:

Brawne, which Tooke conjectured to be boaren, flesh being understood, was applied anciently in a more general sense than at present. The etymology of the word may be traced with much probability to the Latin, *aprugnum*, *callum*. Piers Ploughman speaks of "brawn and blod of the goos, bacon and colhopes;" and Chaucer in the Knight's Tale applies the word, as it has been here, to the muscular parts of the human frame.

"His limnes gret, his braunes hard and strong."

The gloss on Gautier de Bibelesworth gives the word in this sense,

"*En la jambe est la sure*, (the caalf.)
*E taunt cum braoun rest ensure*. (the brahun.)"—Arund. MS., 220, f. 298.

"Brawne of a man, *musculus*." CATH. ANGL. "*Lacerna*, *vel lacertus*, *proprie*, *superior pars brachii vel musculus*, brawne of the arme." MED. Harl. MS., 2257. "He hath eate all the braune of the lopster, *callum*." HORM. "*Braon*, *le gras des fesses*." ROQUEF. Roman de Rou.

The *Catholicon* gives *sura* as well as *musculus* as an equivalent of "brawne of a man," and has also "**Brawne**; *aprina*, *pulpa*; *ap*rinus, *pulpo*sus." Mr. Herrtage's notes are:

"*Musculus*. A muscle or fleashie parte of the bodie compacte of fleash, veines, sinewes and arteries, seruyng especially to the motion of some parte of the bodie by means of the sinewes in it. *Musculosus*. Harde and stiffe with many muscles or brawnes of harde and compacte fleash." Cooper. Chaucer, in the Prologue to the Canterbury Tales, 546, tells us that "The Mellere was a stout carl for the nones,
Ful big he was of *braun*, and eek of boones."

and in the Legende of Goode Women, Dido, l. 145, Eneas is described as of
"a noble visage for the noones,
And formed wel of *brawnes* and of boones."

Cooper gives "*Pulpa*. The woodde of all trees that may be seperated or clefte by the grayne of it, and is the same in timber that *musculus* is in a mans bodie. A muscle or fleashie parte in the bodie of man or beaste. A peece of fleash." "*Pulpa*. Brawne." Medulla. O. Fr. *braon*.

See the *Song of Roland*, l. 97, where the boar is described as tearing a man's arm "clene from the *braun*, the flesche, & the lier."

In the *Sege of Melayne*, 1599, the provisions of the French army are said to have been "brede, *brawne* and wyne." See the Babees Book, p. 53.

The *New English Dictionary* gives from *Piers Plowman* (xiii. 62) the variant reading (Crowley Text) of the line quoted by Mr. Way: "Wombe-cloutes and wylde braune & egges yfryed with grece." These with "mortrewes ['a kind of soup,'] and puddynges" are represented as forming a specially good meal. "Wombe-cloutes" were pieces of omentum (see Wright's *Vocab.*, 789, 19), and now known as tripe, which it will be remembered Mr. Filer with such statistical dogmatism pronounced to be a most wasteful article of consumption. "Wombe-cloutes" are, as Prof. Skeat says, literally "belly-rags." Womb signified the whole or any part of the abdomen. Jack Falstaff, bemoaning his corpulence, uttered the pathetic lament, "My womb, my womb, my womb undoes me." (2 *Henry IV.*, iv. 3, 25). "Cloutes" originally meant bits or pieces of anything,

